# Analysis of in vitro ADCC and clinical response to trastuzumab: possible relevance of FcγRIIIA/FcγRIIA gene polymorphisms and HER-2 expression levels on breast cancer cell lines

**DOI:** 10.1186/s12967-015-0680-0

**Published:** 2015-10-08

**Authors:** Silvia Boero, Anna Morabito, Barbara Banelli, Barbara Cardinali, Beatrice Dozin, Gianluigi Lunardi, Patrizia Piccioli, Sonia Lastraioli, Roberta Carosio, Sandra Salvi, Alessia Levaggi, Francesca Poggio, Alessia D’Alonzo, Massimo Romani, Lucia Del Mastro, Alessandro Poggi, Maria Pia Pistillo

**Affiliations:** Unit of Molecular Oncology and Angiogenesis, IRCCS AOU San Martino-IST, Genoa, Italy; Unit of Tumor Epigenetics, IRCCS AOU San Martino-IST, Genoa, Italy; Development of Innovative Therapies Unit, IRCCS AOU San Martino-IST, Genoa, Italy; Clinical Epidemiology Unit, IRCCS AOU San Martino-IST, Genoa, Italy; Medical Oncology Unit, Sacro Cuore Don Calabria Hospital, Negrar, Verona, Italy; Cellular Biology Unit, IRCCS AOU San Martino-IST, Genoa, Italy; Laboratory of Molecular Diagnostics, IRCCS AOU San Martino-IST, Genoa, Italy; Unit of Pathology, IRCCS AOU San Martino-IST, Genoa, Italy; Unit of Medical Oncology 2, IRCCS AOU San Martino-IST, Genoa, Italy

**Keywords:** FcγR single nucleotide polymorphisms, ADCC, Trastuzumab, Breast cancer patients

## Abstract

**Background:**

Trastuzumab is a humanized monoclonal antibody (mAb) currently used for the treatment of breast cancer (BC) patients with HER-2 overexpressing tumor subtype. Previous data reported the involvement of FcγRIIIA/IIA gene polymorphisms and/or antibody-dependent cellular cytotoxicity (ADCC) in the therapeutic efficacy of trastuzumab, although results on these issues are still controversial. This study was aimed to evaluate in vitro the functional relationships among FcγRIIIA/IIA polymorphisms, ADCC intensity and HER-2 expression on tumor target cells and to correlate them with response to trastuzumab.

**Patients and methods:**

Twenty-five patients with HER-2 overexpressing BC, receiving trastuzumab in a neoadjuvant (NEO) or metastatic (MTS) setting, were genotyped for the FcγRIIIA 158V>F and FcγRIIA 131H>R polymorphisms by a newly developed pyrosequencing assay and by multiplex Tetra-primer-ARMS PCR, respectively. Trastuzumab-mediated ADCC of patients’ peripheral blood mononuclear cells (PBMCs) was evaluated prior to therapy and measured by ^51^Chromium release using as targets three human BC cell lines showing different levels of reactivity with trastuzumab.

**Results:**

We found that the FcγRIIIA 158F and/or the FcγRIIA 131R variants, commonly reported as unfavorable in BC, may actually behave as ADCC favorable genotypes, in both the NEO (*P* ranging from 0.009 to 0.039 and from 0.007 to 0.047, respectively) and MTS (*P* ranging from 0.009 to 0.032 and *P* = 0.034, respectively) patients. The ADCC intensity was affected by different levels of trastuzumab reactivity with BC target cells. In this context, the MCF-7 cell line, showing the lowest reactivity with trastuzumab, resulted the most suitable cell line for evaluating ADCC and response to trastuzumab. Indeed, we found a statistically significant correlation between an increased frequency of patients showing ADCC of MCF-7 and complete response to trastuzumab in the NEO setting (*P* = 0.006).

**Conclusions:**

Although this study was performed in a limited number of patients, it would indicate a correlation of FcγR gene polymorphisms to the ADCC extent in combination with the HER-2 expression levels on tumor target cells in BC patients. However, to confirm our findings further experimental evidences obtained from a larger cohort of BC patients are mandatory.

**Electronic supplementary material:**

The online version of this article (doi:10.1186/s12967-015-0680-0) contains supplementary material, which is available to authorized users.

## Background

Leukocyte Fcγ receptors (FcγRs) are structurally related glycoproteins belonging to the immunoglobulin (Ig) superfamily, which bind the fragment C (Fc) region of IgG leading to FcγR aggregation at the cell surface and cell activation [1]. FcγRs are divided into 3 groups, FcγRI (CD64), FcγRII (CD32) and FcγRIII (CD16) and subclasses, depending on their structural and functional properties. FcγRI and FcγRII are expressed mainly by monocytes and macrophages, whereas FcγRIII is expressed by subsets of T cells and natural killer cells [2].

The most important function of the FcγRs is the regulation of the immune response as, upon their engagement, they can either stimulate or inhibit immune cell activation [2], suggesting that they can have a biological significance in the outcome of antibody-based therapies in tumors.

Among the functions mediated by activatory FcγRs, the antibody-dependent cellular cytotoxicity (ADCC), mediated by cells of the innate immunity, certainly plays an important role as it can contribute to eliminate tumor cells and, in some tumors, it may even represent the predominant effector of cell killing [3–6].

In the immunotherapy of HER-2 overexpressing BC, trastuzumab monoclonal antibody (mAb) may elicit ADCC upon binding to the HER-2 antigen region on tumor cells and with the Fc region to FcγRs on immune effector cells [7, 8]. This mechanism is similar to that of other mAbs of the IgG1 isotype, such as rituximab and cetuximab, upon binding to CD20 and EGFR, respectively [9–12]. Such an interaction triggers the immune cell degranulation and activation, resulting in the lysis of antibody coated target cells.

However, only 20–30 % of HER-2-overexpressing patients respond to trastuzumab and a significant proportion of them become resistant to the mAb [13].

The extent of in vitro ADCC of BC cells mediated by trastuzumab may be influenced by several factors including single nucleotide polymorphisms (*SNPs*) in the FcγR genes. It has been reported that these *SNPs* can alter the FcγR binding to the therapeutic mAbs and consequently the ADCC degree. In particular, the *SNP* rs396991 (G>T) corresponding to the substitution of valine (V) with phenylalanine (F) at aminoacid position 158 of FcγRIIIA (158V>F variant) and the *SNP* rs1801274 (A>G) corresponding to the substitution of histidine (H) with arginine (R) at aminoacid position 131 of FcγRIIA (131H>R variant), appear to reduce the binding to the mAbs [14–16].

However, the association between FcγR polymorphisms and trastuzumab efficacy in BC is controversial. Indeed, the homozygous FcγRIIIA158V/V and FcγRIIA 131H/H phenotypes (commonly identified as 158V/V and 131H/H genotypes) have been associated with ADCC, response to trastuzumab and progression-free survival in two small retrospective studies [7, 17], whereas a larger study did not support these findings [18].

In the present study, we have investigated the FcγRIIIA158V>F and FcγRIIA131H>R genotype frequencies in patients with BC overexpressing HER-2 and their role in the extent of in vitro trastuzumab-dependent lysis of HER2-positive BC cells. We demonstrate that PBMCs from BC patients carrying the FcγRIIIA158F genotype can induce, in some circumstances, a more efficient ADCC response than PBMCs carrying the homozygous FcγRIIIA 158V/V genotype. We also demonstrate that the ADCC associated to particular FcγRIIIA and FcγRIIA genotypes can be influenced by the HER-2 expression levels on target cells. In this context MCF-7, a BC cell line showing the lowest HER-2 expression level, allowed us to point out a correlation between genotypes and ADCC, as well as between ADCC and patient response to trastuzumab.

## Methods

### Patients

Women with histological diagnosis of locally advanced invasive or metastatic BC were considered eligible for the study if classified as HER-2 positive, i.e. score 3+ (by immuno-histochemical analysis: IHC) or IHC score 2+ and FISH (fluorescence in situ hybridization) amplified. Twenty-five BC patients were enrolled in the study: 15 patients in the neo-adjuvant setting (NEO) and 10 patients in the metastatic setting (MTS).

In the NEO setting, all patients (with the exclusion of 1 treated only with paclitaxel) were treated with FEC (fluorouracil, epirubicin and cyclophosphamide) for 4 cycles followed by weekly paclitaxel for 12 weeks in combination with trastuzumab. In the MTS setting, patients underwent a first line chemotherapy in combination with trastuzumab.

Response to trastuzumab was evaluated on the basis of clinical, pathological and radiologic examination of the tumor before and after treatment. In details, for the NEO patients, pathological complete response (pCR) was used to evaluate the treatment response. pCR was assigned in absence of invasive residual carcinoma in the breast and/or at axillary lymph node level after surgery. In the presence of residual invasive carcinoma the response was considered partial (pPR). For the MTS patients, the revised RECIST criteria (version 1.1) were used to evaluate the treatment response which was classified as stable disease (SD), partial response (PR), complete response (CR) and disease progression (PD).

This study was approved by the Ethics Committee of IRCCS AOU San Martino-IST, Genoa, Italy and written informed consent was obtained from each patient.

Thirty-three unrelated healthy Italian women (Transfusion Service, Galliera Hospital, and IRCCS AOU San Martino-IST, Genoa, Italy), matched for patient’s age, were also included as a control population, upon written informed consent. In addition, a cohort of 64 unselected healthy donors were analyzed for FcγRIIIA and FcγRIIA genotyping and ADCC.

### Genotyping of FcγRIIIA 158V>F and FcγRIIA 131H>R polymorphisms

Genotyping of FcγRIIIA 158V>F (rs396991) and FcγRIIA 131H>R (rs1801274) variants was performed on genomic DNA by multiplex Tetra-Primer Amplification Refractory Mutation System (T-ARMS) PCR, direct sequencing (SBT) and pyrosequencing (PSQ). Genomic DNA was extracted from peripheral blood mononuclear cells (PBMCs) using a conventional proteinase K protocol, as previously described [19].

The FcγRIIIA was analyzed by PSQ and due to the high homology of FcγRIIIA with FcγRIIIB in the genomic region of interest, DNA samples were first amplified by primer pair specifically designed for it. To this aim, the PCR reaction was performed in a final volume of 20 μl containing ≈25 ng genomic DNA, 1.5 mM Mg^2+^ (5x buffer A and buffer B), 200 μmoli/L dNTPs, 0.8 μL Elongase^®^ Enzyme Mix (Life Technologies) and 0.5 μmol/L of each primers (Table 1).

**Table 1:** Primers and conditions for the analysis of FcγRIIIA gene polymorphism

Name^a^	Primer sequence	Amplicon size (bp)	Sequence to analyze
FcγRIIIA-F	AAGTCATTTGGGGTCAATTTC	2416^d^	
FcγRIIIA-R	CAGAATAGTTT*C*ATCTCGTATATC^b^		
FcγRIIIA-R-S	CAGGAATAAGGTGACGGTGG	598	
Py-FcγRIIIA-F	*Bio*-AAAGCCACACTCAAAGA**C**AGC^c^	122^e^	**M**AAGCCCCCTGCAGAAGTAG^f^
Py-FcγRIIIA-R	ATTCCAGGGTGGCACATGTC		
Py-FcγRIIIA-S	ACACATTTTTACTCCCAA		

^a^Genbank reference sequence: NG_009066.1; *F* forward, *R* reverse, *Py* pyrosequencing, *S* sequence, *Bio* biotinylated

^b^In italic, underlined, is indicated the deliberate mismatch, from the reference sequence, introduced to improve primer stability

^c^To increase the specificity of the FcγRIIIA amplification, the pyrosequencing primer was designed to include a cytosine (bold underlined) that is replaced by a thymidine in the FcγRIIIB gene (genebank reference sequence. NG_032926.1)

^d^Cycling conditions: 30″ at 94 °C followed by 35 cycles at 94 °C for 30″, 56 °C for 30″, 68 °C for 2′45″ and a final cycle at 70 °C for 10′

^e^Cycling conditions:10′ at 95 °C followed by 45 cycles at 95 °C for 30″, 57 °C for 30″, 72 °C for 30″ and a final cycle at 70 °C for 10′

^f^The polymorphism, in bold, is reported according to the IUB (International Union of Biochemistry) nomenclature

PCR products were analyzed on 1 % agarose gel containing Eurosafe Nucleic Acid Stain (EuroClone, Milan, Italy) and the remaining amount was purified and diluted 1:20; then, to confirm specificity of FcγRIIIA amplification, a short amplicon was analyzed by direct sequencing (using the FcγRIIIA-F and FcγRIIIA-R-S primers (Table 1).

For the PSQ assay, a nested-PCR was setup by amplifying 1 μl of diluted PCR products with the primer pair designed using the Pyrosequencing Assay Design software (Biotage, Uppsala, Sweden) (Table 1).

The PSQ-PCR reactions were assembled with the EpMotion5070 liquid handling station (Eppendorf, Milan, Italy) in a final volume of 50 μl containing 200 µmol/l dNTPs, 1× GeneAmp buffer (1.5 mM MgCl_2_), 1.25U of Immolase Hot Start polymerase (Bioline, Milan, Italy) and 0.3 μM of the PCR primer pairs specific for the FcγRIIIA 158V>F *SNP*. The Pyro Gold reagent kit PSQ 96MA was used for the sequencing reactions, according to the manufacturer instructions. The PSQ assay was performed using a PSQ96MA instrument (Qiagen, Milan, Italy) and the sequencing analysis was conducted with the PSQTM 96MA (version 2.02) software.

The FcγRIIA 131H>R *SNP* was analyzed by T-ARMS PCR [20] and by PCR amplification followed by SBT [7] in both forward and reverse directions using previously described methods.

Both FcγRIIIA 158V>F and FcγRIIA 131H>R polymorphisms were independently re-tested in a different laboratory by T-ARMS PCR or SBT, as previously described, and results where fully concordant.

### Cell lines and culture conditions

The BC cell lines SKBR3, BT474 and MCF-7 (American Type Culture Collection; ATCC, Rockville, MD, USA) were maintained in monolayer culture in RPMI 1640 medium supplemented with 10 % heat-inactivated FBS, 5 mg/ml penicillin and 5 mg/ml streptomycin, 2 mM l-glutamine, at 37 °C in a humidified 5 % CO_2_ atmosphere and subcultured every 3–7 days. The leukaemic cell line K562 (ATCC) was grown in complete RPMI 1640 culture medium. All reagents were purchased from Biochrom KG (Berlin, Germany).

### Analysis of trastuzumab reactivity by flow cytometry

For HER-2 cell surface staining with trastuzumab antibody (Herceptin; Roche, Basel, Switzerland), titration assays were performed by indirect immunofluorescence using as targets SKBR3, BT474 and MCF-7 cell lines. In particular, 2 × 10^5^ cells/sample were incubated with different concentrations (2, 0.2, 0.02, 2 × 10^−3^ and 2 × 10^−4^ µg/ml) of the mAb for 30 min at 4 °C. After washing in FACS buffer, an Alexafluor 647-conjugated goat anti-human IgG secondary antibody (Molecular Probes, Inc. Eugene, OR, USA) was added and incubated for 30 min at 4 °C. After 2 washes, the cells were analyzed by flow cytometry on a CyAn-ADP-Flow-Cytometer (Beckman-Coulter); 5000 cellular events were analyzed per sample. Data were analyzed using Summit version 4.3 Software (Beckman-Coulter).

Results were expressed as mean ratio of relative fluorescence intensity (MRFI), calculated as follows: mean fluorescence intensity (MFI) of HER-2 staining/MFI of secondary antibody staining.

### PBMC separation and antibody-dependent cellular cytotoxicity (ADCC) assay

PBMCs were obtained after Ficoll-Hypaque density centrifugation of blood samples derived from BC patients before starting trastuzumab treatment. In particular, the NEO PBMCs included 4 V/V carriers, 9 V/F or F/F carriers for the FcγRIIIA *SNP* and 3 H/H carriers, 10 H/R or R/R carriers for the FcγRIIA *SNP*. The MTS PBMCs included 3 V/V carriers, 6 V/F or F/F carriers for the FcγRIIIA *SNP* and 4 H/H carriers, 5 H/R or R/R carriers for the FcγRIIA *SNP*.

Ex-vivo isolated PBMCs were used as effector cells in the ADCC assay in the presence of trastuzumab. As in preliminary experiments we found that ADCC triggering could be effective in a wide range of trastuzumab concentrations (from 2 to 2 × 10^−4^ µg/ml), we chose to perform all the cytolytic assays with trastuzumab at 2 µg/ml (concentration at which the differences in trastuzumab reactivity among target cell lines were more evident by flow cytometry). SKBR3, BT474 and MCF-7 target cells were labelled with sodium ^51^Chromate (^51^Cr) and seeded in 96-V-bottomed microplates with effector cells (E)/target (T) at different E:T ratios in 200 µl of volume. Triplicate wells were set up for each E:T ratio and the percentage of lysis was calculated as previously described [21]. In details, the percent of specific cell lysis was calculated as [(experimental − spontaneous release)/(maximum − spontaneous release)] × 100, in which spontaneous release refers to the ^51^Cr release of target cells incubated for 4 h without effector cells. The maximum release is the ^51^Cr release of each target cell in the presence of 1 N HCl. The maximum lysis was represented by the maximum − spontaneous release.

K562 cells were also used as HER-2 negative target cells. Basal cell lysis and ADCC were scored as present (≥5 % of lysis) or absent (<5 % of lysis). We estimated variability among the PBMC lytic activities by the coefficient of variation (CV %), calculated as the standard deviation of cell lysis values among all the PBMC samples tested divided by the mean and multiplied by 100.

### Statistical analysis

Deviation from the Hardy–Weinberg Equilibrium (HWE) was analyzed with the Pearson Chi square test by using the de Finetti program (http://ihg2.helmholtz-muenchen.de/cgi-bin/hw/hwa1.pl). A *P* value <0.05 indicates a lack of HWE.

The comparison of genotype and allele frequencies between groups, as well as the association between genotype frequency, clinical-pathological parameters and response to trastuzumab were performed by the Pearson Chi square test or the Fisher’s exact test, as appropriate. For the correlation test between response to trastuzumab and cytotoxicity (both basal and trastuzumab-mediated), the Bonferroni correction for three comparisons (indicated as *P*^*c*^), that would require a P value <0.017 to reject the null hypothesis, was applied. The comparison between basal cell lysis and trastuzumab-mediated lysis was performed using a paired Student’s t test.

All statistical tests were two-sided and they were carried out using the SPSS package (version 19.0 for Windows). Statistical significance was accepted for any *P* value <0.05.

## Results

### Frequencies of FcγRIIIA and FcγRIIA polymorphisms in HER-2 positive BC patients and in control subjects

In this study, we analysed FcγRIIIA 158V>F and FcγRIIA 131H>R variants in 58 Italian subjects including 25 HER-2 positive BC patients. Out of these latter, 15 were in the neoadjuvant (NEO) setting and 10 in the metastatic (MTS) setting. Thirty-three healthy control women (CTR), matched for age, were also investigated. The main characteristics of patients are summarized in Table 2.

**Table 2:** Patient characteristics and response to trastuzumab

	NEO setting *n* (%)	MTS setting *n* (%)
Number of patients	15 (100)	10 (100)
Median age, years (range)	44.1 (29–81)	57.4 (40–91)
≤50	8 (53.3)	4 (40.0)
>50	7 (46.7)	6 (60.0)
Menopausal status		
Pre	8 (53.3)	4 (40.0)
Post	7 (46.7)	6 (60.0)
Hormonal status		
Either ER+ or PgR+	8 (53.3)	6 (60.0)
ER−/PgR−	7 (46.7)	4 (40.0)
Number of metastatic sites		
1	–	5 (50.0)
≥2	–	5 (50.0)
Metastatic sites		
Non visceral	–	3 (30.0)
Visceral*	–	7 (70.0)
Response to trastuzumab		
Complete response	12 (80.0)	3 (30.0)
Partial response	3 (20.0)	4 (40.0)
Stable disease	–	3 (30.0)

*NEO* neoadjuvant, *MTS* metastatic, *ER* estrogen receptor, *PGR* progesterone receptor (the cut-off value for ER and PGR positivity was ≥1 % of tumor cells with nuclear staining)

* Visceral with or without non visceral metastatic sites

NEO and MTS patients showed similar frequencies of FcγRIIIA genotypes (*P* = 0.763), as well as of alleles (*P* = 0.815; data not shown), as previously reported [17].

Regarding the FcγRIIA *SNP*, the NEO patients showed similar allele frequencies as compared to the MTS patients (*P* = 0.817, data not shown) but a lower frequency of both homozygous genotypes and a higher frequency of the heterozygous genotype (20 *vs* 40 % H/H, 26.7 *vs* 40 % R/R and 53.3 *vs* 20 % H/R), although this difference was not statistically significant (Table 3). The frequency of FcγRIIIA and FcγRIIA genotypes and alleles of BC patients did not significantly differ from those of healthy age-matched CTR when considered either separately (NEO *vs* CTR genotypes, *P* = 0.751 and *P* = 0.836, respectively; NEO *vs* CTR alleles, *P* = 0.716 and *P* = 0.563, respectively); MTS *vs* CTR genotypes, *P* = 0.416 and *P* = 0.202, respectively; MTS *vs* CTR alleles, *P* = 0.961 and *P* = 0.812, respectively) or grouped together with CTR, as reported in Table 3.

**Table 3:** Genotypic and allelic frequencies of FcγRIIIA and FcγRIIA polymorphisms in breast cancer patients and healthy controls

Genotypes	*n* (%)			P*	Alleles	*n* (frequency)			*P°*
	NEO (*n* = 15)	MTS (*n* = 10)	CTR (*n* = 33)			NEO (2*n* = 30)	MTS (2*n* = 20)	CTR (2*n* = 66)	
FcγRIIIA 158V>F					FcγRIIIA 158V>F				
V/V	4 (26.7)	3 (30.0)	6 (18.2)	0.741	V	13 (0.43)	8 (0.40)	26 (0.39)	0.934
V/F	5 (33.3)	2 (20.0)	14 (42.4)		F	17 (0.57)	12 (0.60)	40 (0.60)	
F/F	6 (40.0)	5 (50.0)	13 (39.4)						
HWE	*P* = *0.213*	*P* = *0.065*	*P* = *0.522*						
FcγRIIA 131H>R					FcγRIIA 131H>R				
V/V	3 (20.0)	4 (40.0)	9 (27.3)	0.499	H	14 (0.47)	10 (0.50)	35 (0.53)	0.843
V/F	8 (53.3)	2 (20.0)	17 (51.5)		R	16 (0.53)	10 (0.50)	31 (0.47)	
F/F	4 (26.7)	4 (40.0)	7 (21.2)						
HWE	*P* = *0.782*	*P* = *0.058*	*P* = *0.845*						

Genotyping of FcγRIIIA 158V>F was performed by a newly developed PSQ method after pre-amplification of FcγRIIIA gene. Genotyping of FcγRIIA 131H>R was performed by T-ARMS PCR and SBT. Conventionally, the 158V>F variant corresponds to the G>T *SNP* [i.e. guanine corresponding to valine (V) and thymine corresponding to phenylalanine (F)] and the 131H>R *SNP* corresponds to the A>G *SNP* [i.e. adenine corresponding to histidine (H) and guanine corresponding to arginine (R)]

Comparison of FcγR genotypic and allelic frequencies between patients and control subjects was estimated using the Pearson’s χ^2^ test (P* value) and the Fisher’s test (*P°* value), respectively. Statistical significance: *P* < 0.05

*NEO* neoadjuvant, *MTS* metastatic, *CTR* controls, *HWE* Hardy–Weinberg equilibrium. HWE was tested by the Pearson’s χ^2^ test (*P* < 0.05 indicates lack of HWE)

Control women did not show significantly different FcγRIIIA and FcγRIIA genotype frequencies from those previously reported for the Italian healthy subjects [7, 22]. Moreover, no deviation from the Hardy–Weinberg equilibrium was observed for any of the polymorphisms in BC patients and CTR.

### Analysis of trastuzumab-mediated ADCC in BC patients

**Fig. 1:**
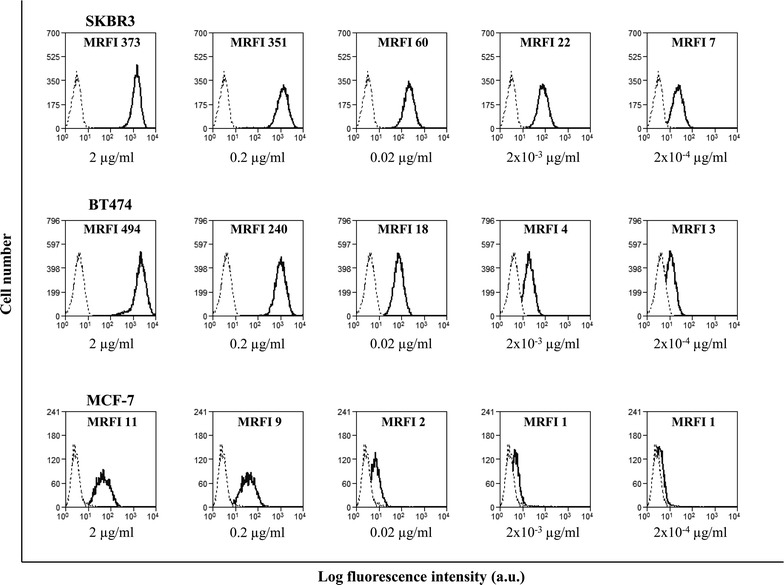
Reactivity of trastuzumab with BC cell lines. Flow cytometric profiles of SKBR-3, BT474 and MCF-7 cell lines stained with serial dilutions (from 2 to 2 × 10^−4^ µg/ml) of trastuzumab in a titration assay. The *solid line* histogram represents the HER-2 staining with trastuzumab and the *dotted line* histogram represents the negative staining with Alexafluor 647-conjugated goat anti-human IgG secondary antibody. *Numbers within the quadrants* represent values of MRFI

We investigated whether PBMCs, ex vivo isolated from NEO and MTS BC patients before receiving trastuzumab, could be activated by the in vitro addition of trastuzumab to elicit ADCC upon interaction with HER-2 expressing BC cells. To this aim, PBMCs were used in a conventional cytolytic assay (at different E:T ratios) against the three BC cell lines SKBR3, MCF-7 and BT474, expressing different levels of reactivity with trastuzumab, as shown by a titration assay using this mAb at different concentrations (from 2 to 2 × 10^−4^ µg/ml). In particular, at 0.02 µg/ml the flow cytometry profiles showed that SKBR3 expressed a high reactivity with trastuzumab, BT474 expressed an intermediate reactivity and MCF-7 expressed a low reactivity with trastuzumab (Fig. 1). This is consistent with previous reports showing SKBR3 as a high HER-2-expressing cell line and MCF-7 as a low or HER-2-negative cell line [23–25].

The cytolytic assays (ADCC) were performed at a constant concentration of 2 µg/ml of trastuzumab or medium alone (basal cytotoxicity) and results were estimated as mean percentage of specific cell lysis. This antibody concentration corresponds to one of the lowest concentration of trastuzumab found in patient serum during treatment (data not shown). Trastuzumab did not elicit ADCC of the HER-2 negative K562 cell line used as internal negative control (data not shown).

**Fig. 2:**
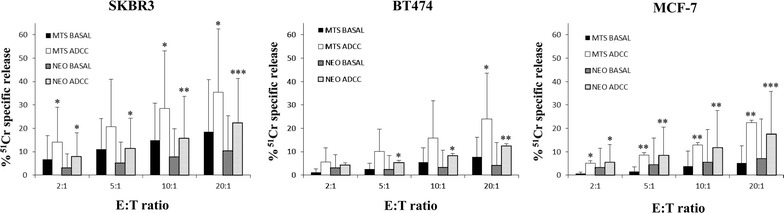
Basal and trastuzumab-mediated cytotoxicity of BC cell lines induced by PBMCs derived from BC patients in the NEO and MTS setting. PBMCs were analyzed for their cytolytic activity in an ADCC assay using trastuzumab (2 µg/ml) and SKBR3, BT474 and MCF-7 cell lines at different effector to target cell ratios (E:T = 2:1, 5:1, 10:1, 20:1). PBMCs were incubated with target cells in medium alone (BASAL) or in the presence of trastuzumab (ADCC). Results are expressed as % of ^51^Cr specific release and are the mean ± SD of experiments with 10 MTS and 15 NEO patients. *NEO* neoadjuvant, *MTS* metastatic, *ADCC* antibody-dependent cellular cytotoxicity. *Asterisks* *on the bars* indicate statistically significant ADCC at the specified E:T ratio evaluated using a paired Student’s t test (see “Results” for *P* values)

The results showed that PBMCs from the MTS patients killed BC cells more efficiently than PBMCs from the NEO patients, at all the E:T ratios both in the absence and in the presence of trastuzumab (Fig. 2). For instance, at 20:1 E:T ratio, the basal cytotoxicity observed for SKBR3, BT474 and MCF-7 was of 18, 8, 5 % for MTS patients and of 10, 4, 7 % for NEO patients, whereas in the presence of trastuzumab the percentage of lysis was 35, 24, 22 % in the MTS cohort compared to 22, 12, 18 % observed in NEO patients, respectively. ADCC was statistically significant at almost all the E:T ratios in both MTS and NEO patients (*P* values ranging from 0.017 to 0.021 with SKBR-3, from 0.002 to 0.012 with MCF-7, *P* value = 0.038 with BT474 for MTS; *P* values at all E:T ratios ranging from <0.001 to 0.010 with all cell lines for NEO). The ADCC degree of NEO and MTS patients mainly reflected the intensity of the cell line reactivity with trastuzumab: i.e. higher ADCC degree was observed with the SKBR3 cell line, followed by the BT474 and the MCF-7 cell lines. This finding was also confirmed in a cohort of healthy donors (not shown).

### Correlation of FcγRIIIA and FcγRIIA genotypes with the ADCC activity of PBMCs from BC patients in the NEO setting

The results of the PBMC-mediated ADCC were then evaluated according to the FcγRIIIA and FcγRIIA gene polymorphisms. Considering the low number of patients within the two BC subgroups, we compared results obtained for the wild type FcγRIIIA 158 V/V and FcγRIIA 131H/H genotypes with those obtained for the mutated 158F (F/V together with F/F) and 131R (R/H together with R/R) carrier genotypes, respectively.

**Fig. 3:**
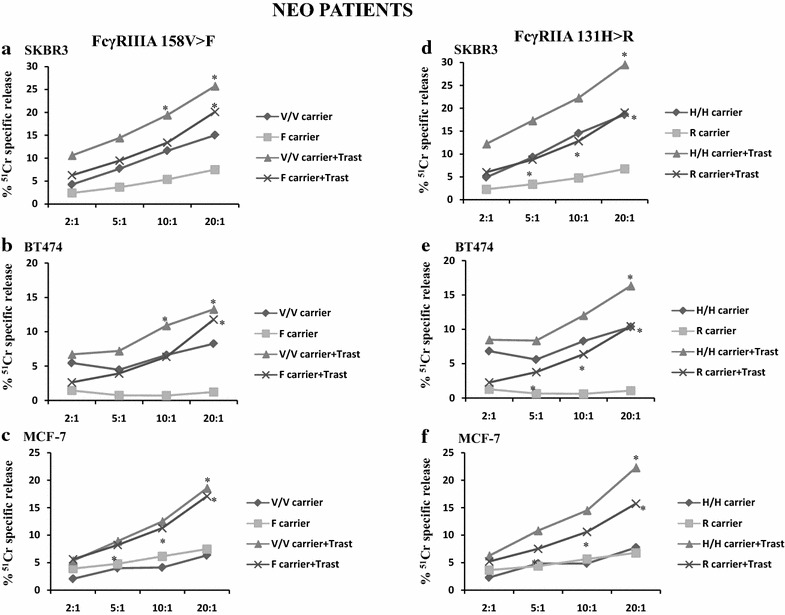
Basal and trastuzumab-mediated cytotoxicity of BC cell lines induced by PBMCs derived from NEO patients according to the FcγRIIIA 158V>F and FcγRIIA 131R>H genotypes. PBMCs from NEO patients were analyzed for their cytolytic activity in an ADCC assay using trastuzumab (2 µg/ml) and SKBR3 (**a**, **d**), BT474 (**b**, **e**) and MCF-7 (**c**, **f**) cell lines at different effector to target cell ratios (E:T = 2:1, 5:1, 10:1, 20:1). PBMCs were incubated with target cells in medium alone (BASAL) or in the presence of trastuzumab (ADCC). Results are expressed as % of ^51^Cr specific release and are the mean of experiments with NEO patients at each E:T ratio. CV % was calculated as the standard deviation of cell lysis values among all the PBMC samples tested divided by the mean and multiplied by 100. CV at 20:1 E:T ratio for SKBR3: ADCC = 86 %, BASAL = 146 %; for BT474: ADCC = 98 %, BASAL = 222 %; for MCF-7: ADCC = 103 %, BASAL = 239 %. **a**–**c** V/V carriers (n = 4) and F carriers (n = 9) refer to PBMCs from patients carrying the FcγRIIIA (158 V/V) wild type genotype and the heterozygous (V/F) together with the mutated (F/F) genotype, respectively, when incubated with target cells in the absence of trastuzumab. Likewise, V/V carriers + Trast and F carriers + Trast, respectively, are refered to PBMCs, showing the genotypes described above, incubated in the presence of trastuzumab. **d**–**f** H/H carriers (n = 3) and R carriers (n = 10) refer to PBMCs from patients carrying the FcγRIIIA (131H/H) wild type genotype and the heterozygous (H/R) together with the mutated (R/R) genotype, respectively, when incubated with target cells in the absence of trastuzumab. Likewise, H/H carriers + Trast and R carriers + Trast, respectively, are referred to PBMCs, showing the genotypes described above, incubated in the presence of trastuzumab. *Asterisks on the curves* indicate significant ADCC at the specified E:T ratio evaluated using a paired Student’s t test (see “Results” for *P* values). *NEO* neoadjuvant, *ADCC* antibody-dependent cellular cytotoxicity, *Trast* trastuzumab

The cytolytic assays performed with the NEO patients showed that trastuzumab triggered PBMCs derived from the FcγRIIIA 158V/V carriers (hereafter 158V/V carriers) to a statistically significant ADCC of SKBR3, BT474 and MCF-7 cell lines at high (20:1) E:T ratio (*P* = 0.022, *P* = 0.042 and *P* = 0.038, respectively; Fig. 3a–c). Significant ADCC was also observed at 10:1 E:T ratio with SKBR3 and BT474 cell lines (*P* = 0.023 and *P* = 0.048, respectively).

PBMCs derived from the FcγRIIIA 158F carriers (hereafter 158F carriers) elicited a statistically significant ADCC of SKBR3 and BT474 cell lines at 20:1 E:T ratio (P = 0.009 and P = 0.016, respectively) and ADCC of MCF-7 also at lower E:T ratios (P = 0.012, P = 0.039 and P = 0.019 at 20:1, 10:1 and 5:1 E:T ratios, respectively) (Fig. 3a–c).

The NEO patients carrying the 158 V/V variant showed a more pronounced ADCC against SKBR3 and BT474 target cell lines at all the E:T ratios, as compared to the 158F carriers, although this difference was not statistically significant (Fig. 3a, b).

The enhancement of basal lysis induced by the 158 V/V carriers was independent from the reduction of HER-2 expression on target cells, whereas the one induced by the 158F carriers was slightly decreasing with the reduction of HER-2 expression (Additional file 1).

Concerning the FcγRIIA polymorphism, PBMCs derived from the FcγRIIA 131H/H carrier (hereafter 131H/H carriers) NEO patients elicited significant ADCC of SKBR3, BT474 and MCF-7 cell lines at an E:T ratio of 20:1 (*P* = 0.010, *P* = 0.029 and *P* = 0.049, respectively; Fig. 3d–f), whereas for PBMCs derived from the 131R carriers (hereafter 131R carriers), significant ADCC of the three cell lines was observed from 20:1 to 5:1 E:T ratios (*P* values ranging from P = 0.007 to P = 0.047; Fig. 3d–f).

The 131H/H carriers elicited higher ADCC degrees as compared to the 131R carriers against all three cell lines, but this difference did not reach statistical significance.

The enhancement of basal lysis induced by the 131H/H carriers was independent from HER-2 expression on target cells, whereas the one induced by the 131R carriers was decreasing with the reduction of HER-2 expression (Additional file 1). Examples of ADCC and basal lysis for each NEO patient are provided against the MCF-7 cell line in Additional file 2.

All these findings demonstrate that, in the NEO patients, both the FcγRIIIA 158F and FcγRIIA 131R carrier genotypes can efficiently induce ADCC also at low E:T ratios and that different HER-2 expression levels on tumor target cells can affect the intensity of ADCC triggered by the FcγRIIIA 158F and 131R carriers.

### Correlation of FcγRIIIA and FcγRIIA genotypes with the ADCC activity of PBMCs from BC patients in the MTS setting

**Fig. 4:**
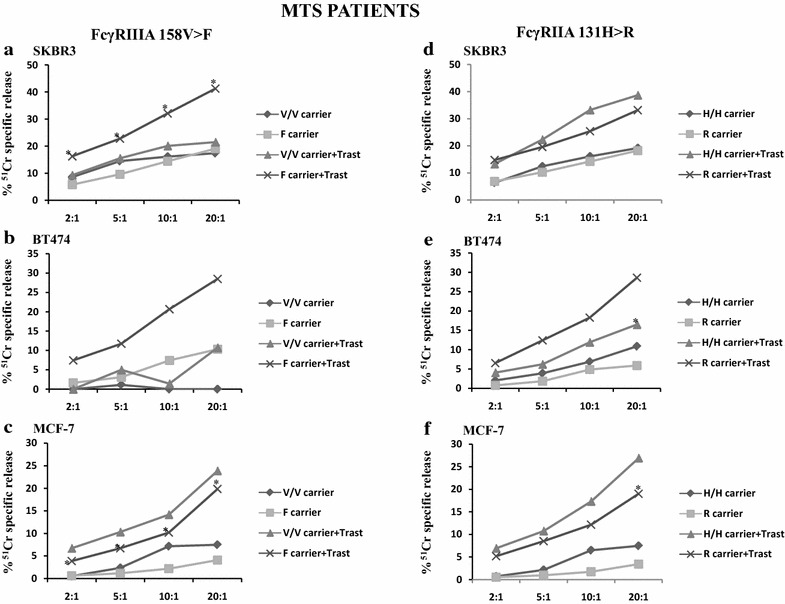
Basal and trastuzumab-mediated cytotoxicity of BC cell lines induced by PBMCs derived from the MTS patients according to the FcγRIIIA 158V>F and FcγRIIA 131R>H genotypes. PBMCs from MTS patients were analyzed for their cytolytic activity in an ADCC assay using trastuzumab (2 µg/ml) and SKBR3 (**a**, **d**), BT474 (**b**, **e**) and MCF-7 (**c**, **f**) cell lines at different effector to target cell ratios (E:T = 2:1, 5:1, 10:1, 20:1). PBMCs were incubated with target cells in medium alone (BASAL) or in the presence of trastuzumab (ADCC). Results are expressed as % of ^51^Cr specific release and are the mean of experiments with MTS patients at each E:T ratio. CV % was calculated as the standard deviation of cell lysis values among all the PBMC samples tested divided by the mean and multiplied by 100. CV at 20:1 E:T ratio for SKBR3: ADCC = 72 %, BASAL = 114 %; for BT474: ADCC = 81 %, BASAL = 108 %; for MCF-7: ADCC = 78 %, BASAL = 140 %. **a**–**c** V/V carriers (n = 3) and F carriers (n = 6) refer to PBMCs from patients carrying the FcγRIIIA (158 V/V) wild type genotype and the heterozygous (V/F) together with the mutated (F/F) genotype, respectively, when incubated with target cells in the absence of trastuzumab. Likewise, V/V carriers + Trast and F carriers + Trast, respectively, are referred to PBMCs, showing the genotypes described above, incubated in the presence of trastuzumab. **d**–**f** H/H carriers (n = 4) and R carriers (n = 5) refer to PBMCs from patients carrying the FcγRIIIA (131H/H) wild type genotype and the heterozygous (H/R) together with the mutated (R/R) genotype, respectively, when incubated with target cells in the absence of trastuzumab. Likewise, H/H carriers + Trast and R carriers + Trast, respectively, are referred to PBMCs, showing the genotypes described above, incubated in the presence of trastuzumab. *Asterisks* *on the curves* indicate significant ADCC at the specified E:T ratio evaluated using a paired Student’s t test (see “Results” for *P* values). *MTS* metastatic, *ADCC* antibody-dependent cellular cytotoxicity, *Trast* trastuzumab

The cytolytic assays performed with the MTS patients show that trastuzumab triggered PBMCs derived from the FcγRIIIA158F carriers to a significant ADCC of SKBR3 and MCF-7 target cells at any of the E:T ratios tested (*P* values ranging from 0.015 to 0.032 and from 0.009 to 0.013, respectively; Fig. 4a, c). In the case of BT474, a trend toward a statistically significant ADCC was observed only at the 20:1 E:T ratio (P = 0.083; Fig. 4b) due to the high variability of ADCC levels observed among patients. In contrast, PBMCs from the 158 V/V carriers did not elicit significant ADCC at any E:T ratio with the cell lines tested.

The 158F carrier PBMCs showed a more pronounced ADCC as compared to the 158V/V carriers, at all the E:T ratios, with SKBR3 and BT474 target cells, but not with MCF-7 target cells (Fig. 4; Additional file 1).

The enhancement of basal lysis induced by the 158F carriers, in the presence of trastuzumab, decreased with the reduction of HER-2 expression on target cells, whereas the one induced by the 158 V/V carriers increased with the reduction of HER-2 surface expression, at any of the E:T ratios (Additional file 1).

Concerning the FcγRIIA polymorphism, PBMCs derived from the 131H/H carrier or 131R carrier MTS patients triggered statistically significant ADCC against BT474 or MCF-7 cell lines at 20:1 E:T ratio (P = 0.028 and P = 0.034, respectively) (Fig. 4e, f). The 131H/H carriers induced a more pronounced ADCC as compared to the 131R carriers against SKBR3 and MCF-7 (Fig. 4d, f), although this difference did not reach statistically significance.

The enhancement of basal lysis induced by both the 131H/H and 131R carriers, in the presence of trastuzumab, was independent from the reduction of HER-2 expression on target cells, at any of the E:T ratios (Additional file 1). Examples of ADCC and basal lysis for each MTS patient are provided against the MCF-7 cell line in Additional file 3.

Taken together, these findings demonstrate that the FcγRIIIA158F carrier genotype can behave as a favourable genotype for promoting ADCC. In addition, different HER-2 expression levels on tumor target cells can affect the intensity of ADCC triggered by the FcγRIIIA 158 V/V and 158F carriers. These findings were confirmed in a cohort of healthy donors (Additional file 4).

### Correlation of basal cytotoxicity and ADCC with prognostic factors and response to trastuzumab

Taking into account that cell cytotoxicity was scored as present (≥5 % of lysis) or absent (<5 % of lysis), we found that, in the NEO patients, the pathological complete response (pCR) to trastuzumab significantly correlated with an increased frequency of patients showing ADCC (Table 4). Indeed, the number of patients with present cytotoxicity in the absence of trastuzumab (BASAL) significantly increased after addition of trastuzumab (from 18.2 to 90.9 % with MCF-7 cell line; *P* = 0.002; Bonferroni corrected *P*^*c*^ = 0.006). A noticeable increase was also observed with BT747 (from 11.1 to 66.7 %) and SKBR3 (from 36.4 to 81.8 %), although it was not statistically significant.

**Table 4:** Correlation of basal cytotoxicity and ADCC with response to trastuzumab of neoadjuvant breast cancer patients

NEO patients	pPR (n = 3)		*P*	pCR (n = 12)		*P*	*P* ^*c*^
	Cytotoxicity			Cytotoxicity			
	*BASAL*	*ADCC*		*BASAL*	*ADCC*		
*SKBR3*	n = 3	n = 3		n = 11	n = 11		
Present	1 (33.3)	3 (100.0)	0.400	4 (36.4)	9 (81.8)	0.080	0.240
Absent	2 (66.7)	0 (–)		7 (63.6)	2 (18.2)		
*BT474*	n = 2	n = 2		n = 9	n = 9		
Present	0 (–)	1 (50.0)	0.400	1 (11.1)	6 (66.7)	0.050	0.147
Absent	2 (100.0)	1 (50.0)		8 (88.9)	3 (33.3)		
*MCF*-*7*	n = 2	n = 2		n = 11	n = 11		
Present	0 (–)	2 (100.0)	0.333	2 (18.2)	10 (90.9)	0.002	0.006
Absent	2 (100.0)	0 (–)		9 (81.8)	1 (9.1)		

PBMCs were analyzed for their cytolytic activity in an ADCC assay using trastuzumab (2 µg/ml) and SKBR3, BT474 and MCF-7 cell lines at 20:1 effector to target cell ratio. PBMCs were incubated with target cells in medium alone (BASAL) or in the presence of trastuzumab (ADCC). Results are expressed as number and percentages (in brackets) of patients showing basal or trastuzumab-mediated cytotoxicity (cut off: >5 %)

Comparison of BASAL cytotoxicity and ADCC was estimated using the Fisher’s test (*P* value) (significance level <0.05) and further adjusted with Bonferroni correction for multiple comparisons (*P* ^*c*^ value) in the indicated patients

*NEO* neoadjuvant, *ADCC* antibody-dependent cellular cytotoxicity, *pPR* pathological partial response, *pCR* pathological complete response

When basal cytotoxicity and ADCC were correlated to the partial response (pPR) no statistically significant results were observed. This analysis was limited by the extremely low number of patients (n = 2 or 3 depending on the cell line used as target; Table 4). Also in the MTS subgroup, the frequency of patients with present cytotoxicity in the CR subgroup increased, upon addition of trastuzumab, when the MCF-7 cell line was used as target (data not shown).

These results suggest that ADCC can play a role in the response to trastuzumab and that this role can be better detectable when using, as target cells, a BC cell line showing low trastuzumab reactivity, such as MCF-7.

We did not find any significant correlation between FcγRIIIA and FcγRIIA gene polymorphisms, basal cell lysis and ADCC with patient age, life status (alive or dead) and prognostic factors including menopausal status, hormonal receptor status, number and sites of metastases, that were found to display significant association with FcγRIIIA/IIA polymorphisms in previous studies [18]. Finally, we did not observe any significant correlations between FcγRIIIA or FcγRIIA *SNPs* and patient response to trastuzumab.

## Discussion

Trastuzumab is a human mAb that specifically binds the extracellular domain of HER-2 receptor expressed by BC cells. Trastuzumab is currently used as a single agent [26] and in combination with chemotherapy [27–29] for the treatment of women with either early [30] or advanced HER-2 overexpressing BC [27, 28]. The principal mechanisms of action of trastuzumab include direct growth inhibition leading to apoptosis of tumor cells, complement-dependent cytotoxicity and ADCC by NK cells, monocytes and granulocytes [31].

It has been previously shown that functional FcγR *SNPs* can regulate the trastuzumab-mediated ADCC and predict the clinical outcome of BC patients treated with trastuzumab-based therapy [7, 17, 32].

In this study, we investigated in vitro the ADCC elicited by PBMCs from NEO and MTS BC patients and correlated its extent with FcγRIIIA 158V>F and FcγRIIA 131H>R genotypes and response to trastuzumab.

We found that the FcγRIIIA 158F and/or the FcγRIIA 131R genotypes, that are commonly reported as unfavourable genotypes not only in BC [7, 32] but also in other tumours [10, 11], may actually behave as favourable genotypes. Indeed, the 158F and 131R genotypes were able to elicit a stronger ADCC, as compared to the 158V/V and 131H/H genotypes, which remained significant also at low E:T ratios. In addition, in some instances, only the 158F and 131R genotypes showed significant ADCC. The MCF-7 cell line has previously been found weakly- or not-expressing HER-2 [24, 25, 33, 34] so that it has been used as a negative control for trastuzumab-mediated ADCC [34]. In our experimental system, MCF-7 displayed low, but detectable, reactivity with trastuzumab by flow cytometry, as compared to SKBR3 and BT474 cell lines that showed high reactivity. Therefore, our ADCC results with MCF-7 cell line may have implications in trastuzumab-based therapy because they suggest that also tumor cells which are not HER-2-amplified and display low levels of HER-2 expression can be efficiently targeted with trastuzumab.

In the NEO patients, the FcγRIIIA 158V/V genotype correlated with a statistically significant ADCC in all cell lines tested, at high E:T ratios, whereas this correlation was not observed in the MTS patients at any E:T ratio. The FcγRIIA131H/H genotype correlated with a statistically significant ADCC at high E:T ratios with two or three cell lines depending on the setting, NEO or MTS, analyzed.

Although different effector cell types within PBMCs can engage trastuzumab, we here chose to evaluate the overall lytic activity of PBMCs isolated before starting trastuzumab therapy in order to mimic the ADCC condition that may occur in vivo. For this reason, we evaluated the FcγRIIIA and FcγRIIA gene polymorphisms that are respectively involved in the activation of the NK cell population and other accessory cells of the immune system. We found a broad interindividual variability of ADCC levels among patients which might be related to the FcγR genotypes, as well as to the proportion and/or lytic efficiency of effector cells present in the PBMCs samples.

The ADCC variability was indicated by the high CV values that we observed among all the PBMC samples tested (CVs ranging from 72 to 103 %, depending on the BC setting and target cell line). Remarkably, we noticed that the variability was more pronounced in the spontaneous (basal) cytotoxicity (CVs ranging from 108 to 239 %), as compared to ADCC, condition that was also observed among healthy individuals (Additional file 4 ). To explain the lower CV values in trastuzumab-mediated cell lysis, it is conceivable that the addition of trastuzumab to the cytolytic assay provides an optimal stimulus to trigger the activation of cytolytic effector cells. Indeed, the FcγRIIIA engagement with trastuzumab resulted in a homogeneous triggering of FcγRIIIA + PBMCs abrogating the individual specific feature of spontaneous lysis of each donor and pointing out their maximal cytolysis. Indeed, only using trastuzumab we could find a biologically significant degree of target cell lysis.

Altogether, the MTS patients showed higher levels of ADCC against all BC cell lines used as targets, as compared to the NEO patients, thus confirming that ADCC can be efficient also in the advanced stage of BC, as reported in previous studies [7, 35]. A possible explanation for the higher ADCC in MTS patients may be the different immunologic context under which immune cells become activated in the two patient settings. In particular, the immune response elicited by BC in the NEO patients might be confined to the tumour microenvironment whereas that elicited in the MTS patients might be systemic resulting from a generalized and more potent immune cell stimulation due to the spread of BC to other parts of the body. Indeed, we found that the ADCC observed in MTS patients was similar, in intensity and variability, to that observed in healthy donors suggesting that MTS patients are in a different activation state as compared to NEO patients. Thus, the different efficacy of MTS and NEO cytolytic activity may be due to intrinsic biological characteristics of the immune cells (activation state, lytic efficiency, different cell number) rather than to the effects of clinical-pathological parameters. In both BC subgroups, the entity of ADCC reflected the HER-2 expression levels as the highest absolute values were observed with the SKBR3 cell line.

In NEO and MTS subgroups the enhancement of basal lysis induced by the FcγRIIIA158F carriers was dependent on HER-2 expression, whereas the one induced by the 158V/V carriers was dependent on HER-2 expression only in the MTS subgroup. Concerning the FcγRIIA, the enhancement of basal lysis induced by the H/H carriers was independent from HER-2 expression in both MTS and NEO subgroups and the one induced by the 131R carrier was dependent on HER-2 expression in NEO, but not in MTS patients.

In summary, these data demonstrate a different behaviour, in respect to the FcγR genotypes and ADCC extent, of BC cell lines expressing different HER-2 levels. These levels might be representative of the tumour and intratumoral heterogeneity of HER-2 expression that has recently been reported in a proportion of breast cancers (36). This heterogeneity is clinically relevant for its association with the response to HER-2-targeted therapy.

To date, there are still conflicting reports in the literature concerning the role of FcγR polymorphisms in the clinical outcome of trastuzumab-treated BC patients. Even the largest retrospective analysis performed by Hurwitz et al. in the adjuvant and metastatic settings does not seem to completely exclude a contribution of FcγR polymorphisms and ADCC/FcγR engagement to the outcome of trastuzumab-treated patients [18]. Thus, the conflicting results may derive from different factors including ethnical differences in the *SNP* frequency, genotyping problems due to the high homology between FcγR members, differences in the modalities of therapeutic use of trastuzumab (i.e. NEO, MTS or adjuvant setting) or in the chemotherapy regimen.

In addition, only few reports analyzed the association of FcγR polymorphisms with ADCC intensity and with clinical outcome [7, 8]; other reports analyzed only the association of FcγR polymorphisms with clinical response without taking into account the ADCC [17, 18, 32], or the association between ADCC and response to trastuzumab without considering the FcγR gene polymorphisms [34, 35]. Although performed in a quite small number of patients, our study correlates FcγR gene polymorphisms to the ADCC extent in combination with the HER-2 expression levels on tumor target cells. Similarly, this study also correlates FcγR polymorphisms to the basal lysis extent. Indeed, in both NEO and MTS patients the 158 V/V carriers showed, in some instances, a more pronounced lysis as compared to that of the 158F carriers, although in MTS patients with BT474 cell line we found that 158F carrier showed a more efficient basal lysis than 158 V/V. However, this difference was not statistically significant and may depend on intrinsic features of each patient.

Our results demonstrate a statistically significant correlation of the ADCC degree with the complete response of NEO patients to trastuzumab. In particular, the frequency of patients with positive ADCC was significantly increased using the MCF-7 cell line as target, as compared to patients without ADCC; this suggests that also small variations in the percent of cell lysis observed in vitro might contribute to the induction of a complete response in vivo.

It is of note that the increase of patients showing cytotoxicity was observed with the MCF-7 cell line suggesting that the use of low-HER-2-expressing cells might be useful for testing of ADCC.

Even if further investigations in larger cohort studies are required to confirm our findings, these results provide additional information for a better understanding of the role of FcγR gene polymorphisms/ADCC in trastuzumab-treated BC; this study might improve the testing of patient ADCC in order to identify subgroups of patients who might benefit from trastuzumab treatment.

## Conclusions

In our experimental system, we could detect that unfavourable FcγRIIIA158F carrier or FcγRIIA131R carrier genotypes may actually behave as favourable genotypes.

Despite the limitation due to the small number of patients enrolled, our findings would suggest that in the in vitro assays for the ADCC activity of patients eligible for trastuzumab therapy it might be useful to include a panel of BC cell lines showing different HER-2 expression levels, as they may reflect the in vivo HER-2 heterogeneity in different tumor areas [36].

## Additional files

10.1186/s12967-015-0680-0 Enhancement of basal lysis of different BC cell lines induced in the presence of trastuzumab.
10.1186/s12967-015-0680-0 Basal and trastuzumab-mediated cytotoxicity of MCF-7 cell line induced by PBMCs derived from the NEO individual patients.
10.1186/s12967-015-0680-0 Basal and trastuzumab-mediated cytotoxicity of MCF-7 cell line induced by PBMCs derived from the MTS individual patients.
10.1186/s12967-015-0680-0 Basal and trastuzumab-mediated cytotoxicity of BC cell lines induced by PBMCs derived from healthy donors.

## Abbreviations

ADCC: antibody-dependent cellular cytotoxicity
BC: breast cancer
FcγR: FcγReceptor
E:T ratio: effector:target ratio
HWE: Hardy–Weinberg Equilibrium
mAb: monoclonal antibody
MRFI: mean ratio of relative fluorescence intensity
MTS: metastatic
NEO: neoadjuvant
PBMCs: peripheral blood mononuclear cells
pCR: pathological complete response
pPR: pathological partial response
PSQ: pyrosequencing
SBT: sequence-based typing
SNPs: single nucleotide polymorphisms
T-ARMS PCR: tetra-primer amplification refractory mutation system PCR
Trast: trastuzumab
