# Erratum to: Analysis of in vitro ADCC and clinical response to trastuzumab: possible relevance of FcγRIIIA/FcγRIIA gene polymorphisms and HER-2 expression levels on breast cancer cell lines

**DOI:** 10.1186/s12967-016-0779-y

**Published:** 2016-01-22

**Authors:** Silvia Boero, Anna Morabito, Barbara Banelli, Barbara Cardinali, Beatrice Dozin, Gianluigi Lunardi, Patrizia Piccioli, Sonia Lastraioli, Roberta Carosio, Sandra Salvi, Alessia Levaggi, Francesca Poggio, Alessia D’Alonzo, Massimo Romani, Lucia Del Mastro, Alessandro Poggi, Maria Pia Pistillo

**Affiliations:** Unit of Molecular Oncology and Angiogenesis, IRCCS AOU San Martino-IST, Genoa, Italy; Unit of Tumor Epigenetics, IRCCS AOU San Martino-IST, Genoa, Italy; Development of Innovative Therapies Unit, IRCCS AOU San Martino-IST, Genoa, Italy; Clinical Epidemiology Unit, IRCCS AOU San Martino-IST, Genoa, Italy; Medical Oncology Unit, Sacro Cuore Don Calabria Hospital, Negrar, Verona, Italy; Cellular Biology Unit, IRCCS AOU San Martino-IST, Genoa, Italy; Laboratory of Molecular Diagnostics, IRCCS AOU San Martino-IST, Genoa, Italy; Unit of Pathology, IRCCS AOU San Martino-IST, Genoa, Italy; Unit of Medical Oncology 2, IRCCS AOU San Martino-IST, Genoa, Italy

## Erratum to: J Transl Med (2015) 13:324 DOI 10.1186/s12967-015-0680-0

It has come the publisher’s attention that the original version of this article [1] unfortunately contained an error. In Table 3, first column, the FcγRIIA 131 H>R genotypes were incorrectly labelled. In particular, V/V should have read H/H, V/F should have read H/R and F/F should have read R/R. Please note that this correction does not change the genotype numerical values of FcγRIIA polymorphism. The correct Table 3 has been published as Table 1 in this Erratum.

**Table 1:** Genotypic and allelic frequencies of FcγRIIIA and FcγRIIA polymorphisms in breast cancer patients and healthy controls

Genotypes	*n* (%)			*P* ^*^	Alleles	*n* (frequency)			*P°*
	NEO (*n* = 15)	MTS (*n* = 10)	CTR (*n* = 33)			NEO (2*n* = 30)	MTS (2*n* = 20)	CTR (2*n* = 66)	
FcγRIIIA 158V>F					FcγRIIIA 158V>F				
V/V	4 (26.7)	3 (30.0)	6 (18.2)	0.741	V	13 (0.43)	8 (0.40)	26 (0.39)	0.934
V/F	5 (33.3)	2 (20.0)	14 (42.4)		F	17 (0.57)	12 (0.60)	40 (0.60)	
F/F	6 (40.0)	5 (50.0)	13 (39.4)						
* HWE*	*P* = *0.213*	*P* = *0.065*	*P* = *0.522*						
FcγRIIA 131H>R					FcγRIIA 131H>R				
H/H	3 (20.0)	4 (40.0)	9 (27.3)	0.499	H	14 (0.47)	10 (0.50)	35 (0.53)	0.843
H/R	8 (53.3)	2 (20.0)	17 (51.5)		R	16 (0.53)	10 (0.50)	31 (0.47)	
R/R	4 (26.7)	4 (40.0)	7 (21.2)						
* HWE*	*P* =* 0.782*	*P* =* 0.058*	*P *= *0.845*						

Genotyping of FcγRIIIA 158V>F was performed by a newly developed PSQ method after pre-amplification of FcγRIIIA gene. Genotyping of FcγRIIA 131H>R was performed by T-ARMS PCR and SBT. Conventionally, the 158V>F variant corresponds to the G>T SNP [i.e. guanine corresponding to valine (V) and thymine corresponding to phenylalanine (F)] and the 131H>R SNP corresponds to the A>G SNP [i.e. adenine corresponding to histidine (H) and guanine corresponding to arginine (R)]

Comparison of FcγR genotypic and allelic frequencies between patients and control subjects was estimated using the Pearson’s χ^2^ test (P* value) and the Fisher’s test (*P*° value), respectively. Statistical significance: *P* < 0.05

*NEO* neoadjuvant, *MTS* metastatic, *CTR* controls, *HWE* Hardy–Weinberg equilibrium. HWE was tested by the Pearson’s χ^2^ test (*P* < 0.05 indicates lack of HWE)
