# Supplementary material for: Analysis of in vitro ADCC and clinical response to trastuzumab: possible relevance of FcγRIIIA/FcγRIIA gene polymorphisms and HER-2 expression levels on breast cancer cell lines
Source: J Transl Med. 2015 Oct 8;13:324. doi: 10.1186/s12967-015-0680-0 (PMC4598965; doi:10.1186/s12967-015-0680-0)
Supplement: Supplementary file 1 — 10.1186/s12967-015-0680-0 Enhancement of basal lysis of different BC cell lines induced in the presence of trastuzumab. [file 12967_2015_680_MOESM1_ESM.docx]

**Additional file: Table S1**. Enhancement of basal lysis of different BC cell lines induced in the presence of trastuzumab. Correlation with FcγRIIIA 158V>F and FcγRIIA 131H>R genotypes in BC patients.

| **Cell line** | **FcγR genotype** | **Delta score at different E:T ratios** | | | | | | | | | | | | | | |
| --- | --- | --- | --- | --- | --- | --- | --- | --- | --- | --- | --- | --- | --- | --- | --- | --- |
|  |  | **NEO** | | | | | | |  | **MTS** | | | | | | |
|  |  | **2:1** |  | **5:1** |  | **10:1** |  | **20:1** |  | **2:1** |  | **5:1** |  | **10:1** |  | **20:1** |
| SKBR3 | ***FcγRIIIA***  158V/V | 6.36 |  | 6.72 |  | 7.80 |  | 10.71 |  | 0.71 |  | 1.03 |  | 3.96 |  | 4.07 |
| BT474 | 158V/V | 1.25 |  | 2.74 |  | 4.28 |  | 5.00 |  | 0.00 |  | 3.87 |  | 1.43 |  | 10.71 |
| MCF-7 | 158V/V | 3.31 |  | 4.91 |  | 8.35 |  | 12.23 |  | 3.76 |  | 6.46 |  | 10.07 |  | 17.76 |
| SKBR3 | 158F* | 3.88 |  | 5.82 |  | 8.04 |  | 12.59 |  | 10.45 |  | 13.19 |  | 17.65 |  | 22.20 |
|  |  |  |  |  |  |  |  |  |  |  |  |  |  |  |  |  |
| BT474 | 158F | 1.19 |  | 3.18 |  | 5.65 |  | 10.55 |  | 5.82 |  | 8.67 |  | 13.22 |  | 18.17 |
| MCF-7 | 158F | 1.67 |  | 3.44 |  | 5.14 |  | 9.61 |  | 6.16 |  | 7.94 |  | 7.00 |  | 16.38 |
|  | ***FcγRIIA*** |  |  |  |  |  |  |  |  |  |  |  |  |  |  |  |
| SKBR3 | 131H/H | 7.28 |  | 7.98 |  | 7.73 |  | 10.91 |  | 6.96 |  | 9.86 |  | 17.16 |  | 19.48 |
| BT474 | 131H/H | 1.65 |  | 2.77 |  | 3.74 |  | 5.98 |  | 2.03 |  | 2.31 |  | 5.01 |  | 5.62 |
| MCF-7 | 131H/H | 3.94 |  | 5.97 |  | 9.65 |  | 14.55 |  | 6.24 |  | 8.62 |  | 10.82 |  | 19.43 |
|  |  |  |  |  |  |  |  |  |  |  |  |  |  |  |  |  |
| SKBR3 | 131R* | 3.74 |  | 5.37 |  | 8.05 |  | 12.28 |  | 7.91 |  | 9.33 |  | 11.15 |  | 14.94 |
| BT474 | 131R | 0.99 |  | 3.12 |  | 5.75 |  | 9.37 |  | 5.77 |  | 10.57 |  | 13.42 |  | 22.71 |
| MCF-7 | 131R | 1.57 |  | 3.16 |  | 4.94 |  | 8.96 |  | 4.65 |  | 7.56 |  | 10.43 |  | 15.59 |

The difference between lysis of a given BC cell line triggered with trastuzumab (2 µg/ml) and basal lysis (i.e. lysis in medium alone) was

defined as delta score. Patients were subdivided into NEO (neoadjuvant) and MTS (metastatic) subgroups. The corresponding genotypes of

FcγRIIIA 158V>F and FcγRIIA 131H>R polymorphisms are indicated on the left.

*158F includes both the 158F/V and 158F/F genotypes; *131R includes both the 131H/R and 131R/R genotypes.
