# Supplementary material for: Analysis of in vitro ADCC and clinical response to trastuzumab: possible relevance of FcγRIIIA/FcγRIIA gene polymorphisms and HER-2 expression levels on breast cancer cell lines
Source: J Transl Med. 2015 Oct 8;13:324. doi: 10.1186/s12967-015-0680-0 (PMC4598965; doi:10.1186/s12967-015-0680-0)
Supplement: Supplementary file 2 — 10.1186/s12967-015-0680-0 Basal and trastuzumab-mediated cytotoxicity of MCF-7 cell line induced by PBMCs derived from the NEO individual patients. [file 12967_2015_680_MOESM2_ESM.pptx]

## Slide 1
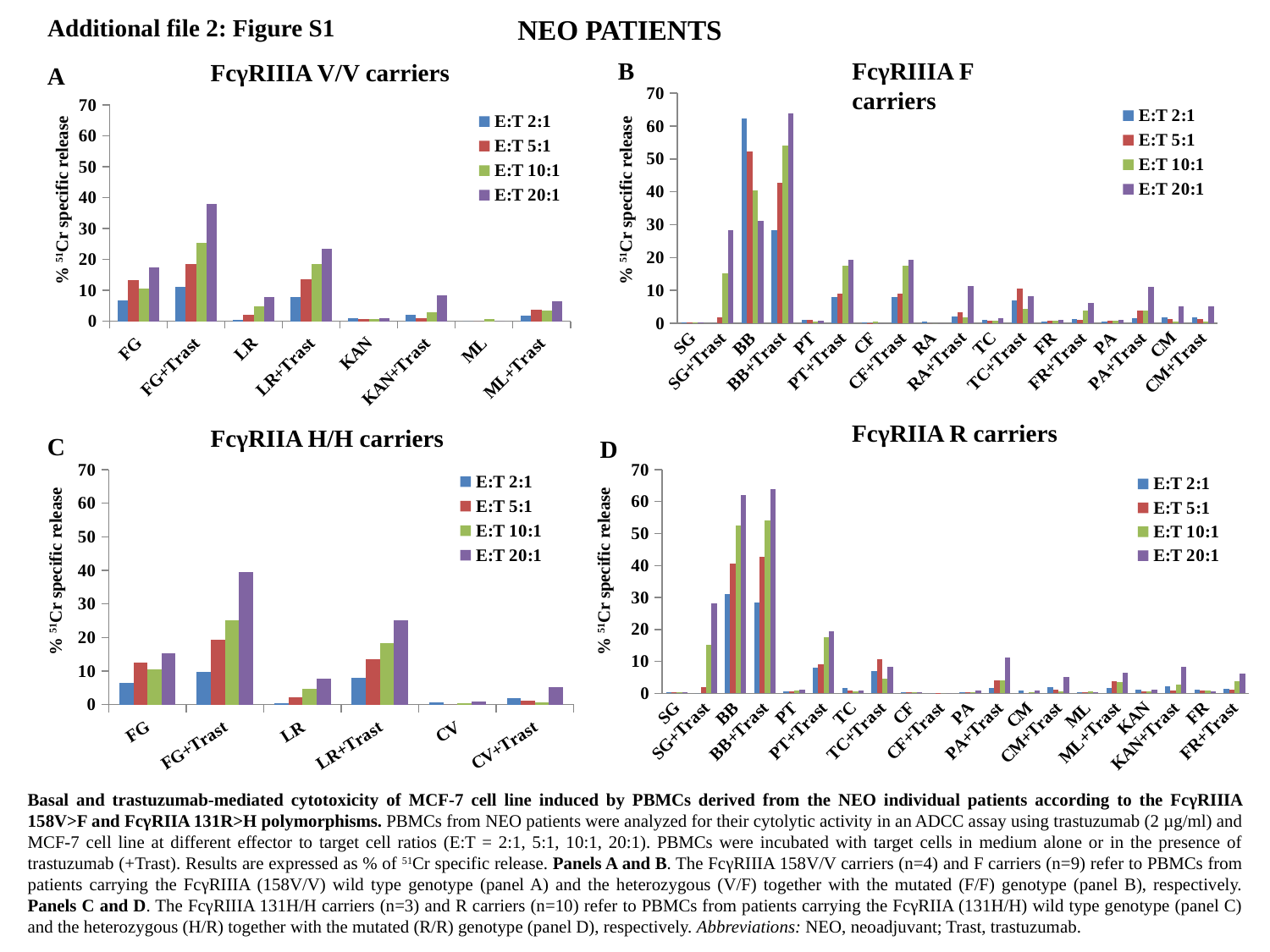

NEO PATIENTS
B
FcγRIIIA F carriers
FcγRIIIA V/V carriers
A
### Chart
| Category | E:T 2:1 | E:T 5:1 | E:T 10:1 | E:T 20:1 |
|---|---|---|---|---|
| SG | 0.25 | 0.25 | 0.25 | 0.25 |
| SG+Trast | 0.0 | 1.82 | 15.2 | 28.29 |
| BB | 62.15 | 52.37 | 40.51 | 31.06 |
| BB+Trast | 28.35 | 42.62 | 54.06 | 63.85 |
| PT | 1.139 | 0.9453 | 0.6137 | 0.6935 |
| PT+Trast | 8.072 | 9.119 | 17.6 | 19.38 |
| CF | 0.3437 | 0.01225 | 0.4572 | 0.0 |
| CF+Trast | 8.072 | 9.119 | 17.6 | 19.38 |
| RA | 0.455 | 0.0 | 0.0 | 0.0 |
| RA+Trast | 2.098 | 3.463 | 1.896 | 11.32 |
| TC | 0.9267 | 0.7148 | 0.807 | 1.654 |
| TC+Trast | 7.012 | 10.6 | 4.489 | 8.383 |
| FR | 0.5309 | 0.8993 | 0.7426 | 1.062 |
| FR+Trast | 1.359 | 1.166 | 3.863 | 6.266 |
| PA | 0.5309 | 0.8993 | 0.7426 | 1.062 |
| PA+Trast | 1.618 | 3.944 | 3.969 | 11.07 |
| CM | 1.949 | 1.24 | 0.6006 | 5.198 |
| CM+Trast | 1.949 | 1.24 | 0.6006 | 5.198 |
### Chart
| Category | E:T 2:1 | E:T 5:1 | E:T 10:1 | E:T 20:1 |
|---|---|---|---|---|
| FG | 6.85 | 13.27 | 10.54 | 17.3 |
| FG+Trast | 11.0 | 18.42 | 25.42 | 38.0 |
| LR | 0.3652 | 2.152 | 4.709 | 7.687 |
| LR+Trast | 7.956 | 13.63 | 18.41 | 23.34 |
| KAN | 1.065 | 0.62 | 0.7241 | 1.092 |
| KAN+Trast | 2.169 | 0.9544 | 2.796 | 8.41 |
| ML | 0.0 | 0.0 | 0.5815 | 0.0 |
| ML+Trast | 1.795 | 3.817 | 3.539 | 6.522 |
% 51Cr specific release
% 51Cr specific release
FcγRIIA R carriers
FcγRIIA H/H carriers
C
D
### Chart
| Category | E:T 2:1 | E:T 5:1 | E:T 10:1 | E:T 20:1 |
|---|---|---|---|---|
| SG | 0.25 | 0.25 | 0.25 | 0.25 |
| SG+Trast | 0.0 | 1.82 | 15.2 | 28.29 |
| BB | 31.06 | 40.51 | 52.37 | 62.15 |
| BB+Trast | 28.35 | 42.62 | 54.06 | 63.85 |
| PT | 0.6935 | 0.6137 | 0.95 | 1.139 |
| PT+Trast | 8.072 | 9.119 | 17.6 | 19.38 |
| TC | 1.654 | 0.807 | 0.71 | 0.9267 |
| TC+Trast | 7.012 | 10.6 | 4.489 | 8.383 |
| CF | 0.45 | 0.4572 | 0.4572 | 0.4572 |
| CF+Trast | 0.0 | 0.1044 | 0.0 | 0.0 |
| PA | 0.2781 | 0.4572 | 0.4572 | 0.7585 |
| PA+Trast | 1.618 | 3.944 | 3.969 | 11.07 |
| CM | 0.7426 | 0.0 | 0.45 | 0.8989 |
| CM+Trast | 1.949 | 1.24 | 0.6006 | 5.198 |
| ML | 0.46 | 0.46 | 0.58 | 0.46 |
| ML+Trast | 1.795 | 3.817 | 3.539 | 6.522 |
| KAN | 1.065 | 0.62 | 0.7241 | 1.092 |
| KAN+Trast | 2.169 | 0.9544 | 2.796 | 8.41 |
| FR | 1.062 | 0.7426 | 0.8993 | 0.5309 |
| FR+Trast | 1.359 | 1.166 | 3.863 | 6.266 |
### Chart
| Category | E:T 2:1 | E:T 5:1 | E:T 10:1 | E:T 20:1 |
|---|---|---|---|---|
| FG | 6.5 | 12.4 | 10.45 | 15.27 |
| FG+Trast | 9.75 | 19.43 | 25.06 | 39.44 |
| LR | 0.3652 | 2.152 | 4.709 | 7.687 |
| LR+Trast | 7.956 | 13.63 | 18.41 | 25.23 |
| CV | 0.7426 | 0.0 | 0.448 | 0.8989 |
| CV+Trast | 1.949 | 1.24 | 0.6006 | 5.198 |% 51Cr specific release
% 51Cr specific release
Basal and trastuzumab-mediated cytotoxicity of MCF-7 cell line induced by PBMCs derived from the NEO individual patients according to the FcγRIIIA 158V>F and FcγRIIA 131R>H polymorphisms. PBMCs from NEO patients were analyzed for their cytolytic activity in an ADCC assay using trastuzumab (2 µg/ml) and MCF-7 cell line at different effector to target cell ratios (E:T = 2:1, 5:1, 10:1, 20:1). PBMCs were incubated with target cells in medium alone or in the presence of trastuzumab (+Trast). Results are expressed as % of 51Cr specific release. Panels A and B. The FcγRIIIA 158V/V carriers (n=4) and F carriers (n=9) refer to PBMCs from patients carrying the FcγRIIIA (158V/V) wild type genotype (panel A) and the heterozygous (V/F) together with the mutated (F/F) genotype (panel B), respectively. Panels C and D. The FcγRIIIA 131H/H carriers (n=3) and R carriers (n=10) refer to PBMCs from patients carrying the FcγRIIA (131H/H) wild type genotype (panel C) and the heterozygous (H/R) together with the mutated (R/R) genotype (panel D), respectively. Abbreviations: NEO, neoadjuvant; Trast, trastuzumab.
Additional file 2: Figure S1
