# Supplementary material for: Analysis of in vitro ADCC and clinical response to trastuzumab: possible relevance of FcγRIIIA/FcγRIIA gene polymorphisms and HER-2 expression levels on breast cancer cell lines
Source: J Transl Med. 2015 Oct 8;13:324. doi: 10.1186/s12967-015-0680-0 (PMC4598965; doi:10.1186/s12967-015-0680-0)
Supplement: Supplementary file 3 — 10.1186/s12967-015-0680-0 Basal and trastuzumab-mediated cytotoxicity of MCF-7 cell line induced by PBMCs derived from the MTS individual patients. [file 12967_2015_680_MOESM3_ESM.pptx]

## Slide 1
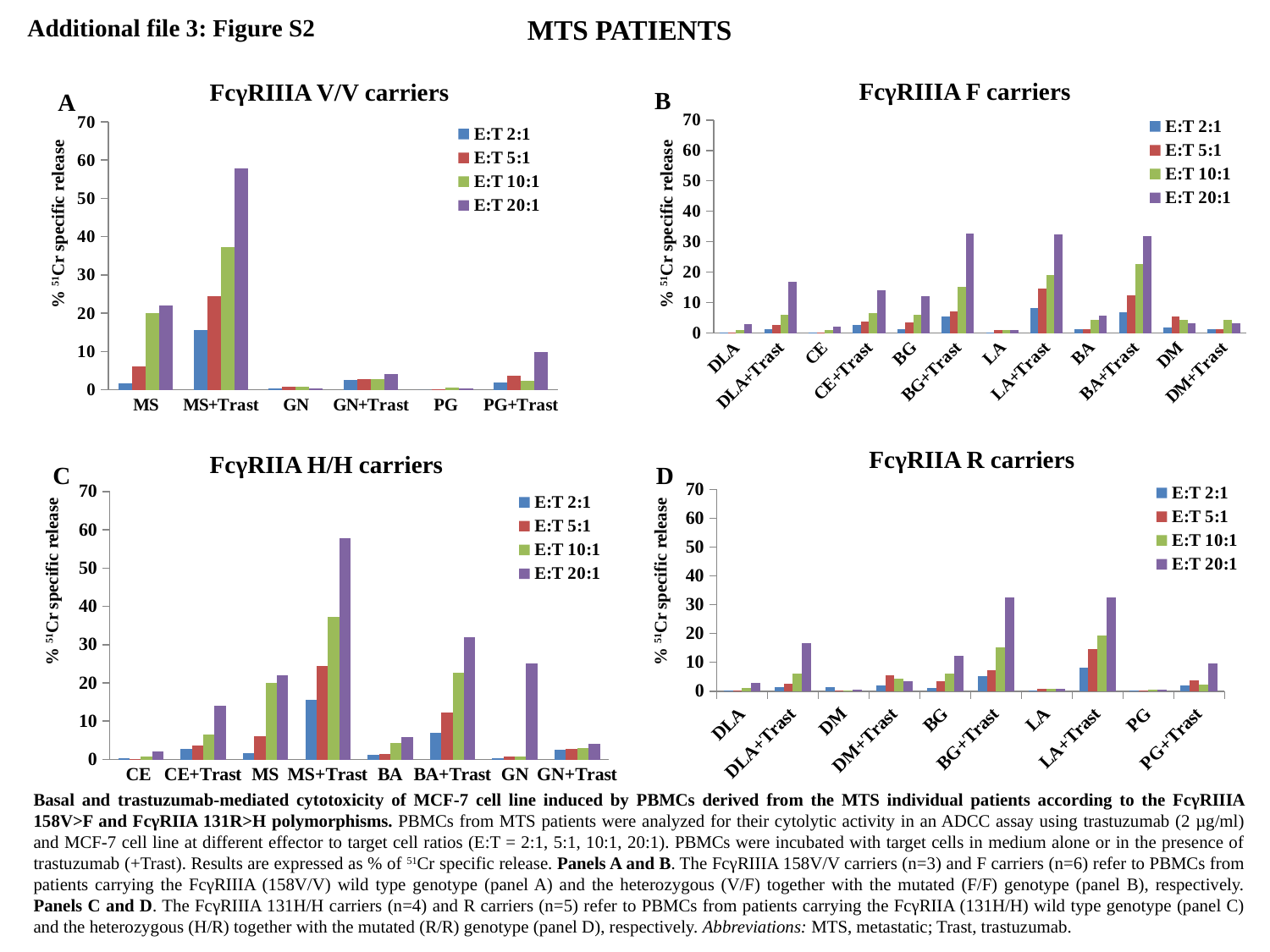

Additional file 3: Figure S2
MTS PATIENTS
FcγRIIIA F carriers
FcγRIIIA V/V carriers
### Chart
| Category | E:T 2:1 | E:T 5:1 | E:T 10:1 | E:T 20:1 |
|---|---|---|---|---|
| DLA | 0.25 | 0.25 | 1.094 | 2.94 |
| DLA+Trast | 1.267 | 2.61 | 5.996 | 16.79 |
| CE | 0.25 | 0.25 | 0.8648 | 2.14 |
| CE+Trast | 2.679 | 3.632 | 6.421 | 13.94 |
| BG | 1.14 | 3.39 | 6.031 | 12.16 |
| BG+Trast | 5.342 | 7.225 | 15.08 | 32.63 |
| LA | 0.25 | 0.9452 | 0.8878 | 0.8878 |
| LA+Trast | 8.281 | 14.58 | 19.16 | 32.5 |
| BA | 1.175 | 1.342 | 4.22 | 5.745 |
| BA+Trast | 6.913 | 12.28 | 22.6 | 31.89 |
| DM | 1.94 | 5.529 | 4.37 | 3.32 |
| DM+Trast | 1.32 | 1.18 | 4.37 | 3.32 |
### Chart
| Category | E:T 2:1 | E:T 5:1 | E:T 10:1 | E:T 20:1 |
|---|---|---|---|---|
| MS | 1.597 | 6.169 | 20.06 | 21.92 |
| MS+Trast | 15.65 | 24.35 | 37.36 | 57.73 |
| GN | 0.45 | 0.8593 | 0.8595 | 0.45 |
| GN+Trast | 2.553 | 2.806 | 2.882 | 4.095 |
| PG | 0.0 | 0.0859 | 0.5493 | 0.4204 |
| PG+Trast | 1.921 | 3.762 | 2.252 | 9.744 |FcγRIIA R carriers
FcγRIIA H/H carriers
### Chart
| Category | E:T 2:1 | E:T 5:1 | E:T 10:1 | E:T 20:1 |
|---|---|---|---|---|
| DLA | 0.25 | 0.25 | 1.094 | 2.94 |
| DLA+Trast | 1.267 | 2.61 | 5.996 | 16.79 |
| DM | 1.32 | 0.3621 | 0.0767 | 0.638 |
| DM+Trast | 1.94 | 5.529 | 4.37 | 3.32 |
| BG | 1.14 | 3.39 | 6.031 | 12.16 |
| BG+Trast | 5.342 | 7.225 | 15.08 | 32.63 |
| LA | 0.176 | 0.9452 | 0.8878 | 0.8878 |
| LA+Trast | 8.281 | 14.58 | 19.16 | 32.5 |
| PG | 0.25 | 0.0859 | 0.5493 | 0.4204 |
| PG+Trast | 1.921 | 3.762 | 2.252 | 9.744 |
### Chart
| Category | E:T 2:1 | E:T 5:1 | E:T 10:1 | E:T 20:1 |
|---|---|---|---|---|
| CE | 0.25 | 0.2105 | 0.8648 | 2.14 |
| CE+Trast | 2.679 | 3.632 | 6.421 | 13.94 |
| MS | 1.597 | 6.169 | 20.06 | 21.92 |
| MS+Trast | 15.65 | 24.35 | 37.36 | 57.73 |
| BA | 1.175 | 1.342 | 4.22 | 5.745 |
| BA+Trast | 6.913 | 12.28 | 22.6 | 31.89 |
| GN | 0.25 | 0.8593 | 0.8595 | 25.0 |
| GN+Trast | 2.553 | 2.806 | 2.882 | 4.095 |
B
A
% 51Cr specific release
% 51Cr specific release
C
D
% 51Cr specific release
% 51Cr specific release
Basal and trastuzumab-mediated cytotoxicity of MCF-7 cell line induced by PBMCs derived from the MTS individual patients according to the FcγRIIIA 158V>F and FcγRIIA 131R>H polymorphisms. PBMCs from MTS patients were analyzed for their cytolytic activity in an ADCC assay using trastuzumab (2 µg/ml) and MCF-7 cell line at different effector to target cell ratios (E:T = 2:1, 5:1, 10:1, 20:1). PBMCs were incubated with target cells in medium alone or in the presence of trastuzumab (+Trast). Results are expressed as % of 51Cr specific release. Panels A and B. The FcγRIIIA 158V/V carriers (n=3) and F carriers (n=6) refer to PBMCs from patients carrying the FcγRIIIA (158V/V) wild type genotype (panel A) and the heterozygous (V/F) together with the mutated (F/F) genotype (panel B), respectively. Panels C and D. The FcγRIIIA 131H/H carriers (n=4) and R carriers (n=5) refer to PBMCs from patients carrying the FcγRIIA (131H/H) wild type genotype (panel C) and the heterozygous (H/R) together with the mutated (R/R) genotype (panel D), respectively. Abbreviations: MTS, metastatic; Trast, trastuzumab.
