# Supplementary material for: Analysis of in vitro ADCC and clinical response to trastuzumab: possible relevance of FcγRIIIA/FcγRIIA gene polymorphisms and HER-2 expression levels on breast cancer cell lines
Source: J Transl Med. 2015 Oct 8;13:324. doi: 10.1186/s12967-015-0680-0 (PMC4598965; doi:10.1186/s12967-015-0680-0)
Supplement: Supplementary file 4 — 10.1186/s12967-015-0680-0 Basal and trastuzumab-mediated cytotoxicity of BC cell lines induced by PBMCs derived from healthy donors. [file 12967_2015_680_MOESM4_ESM.pptx]

## Slide 1
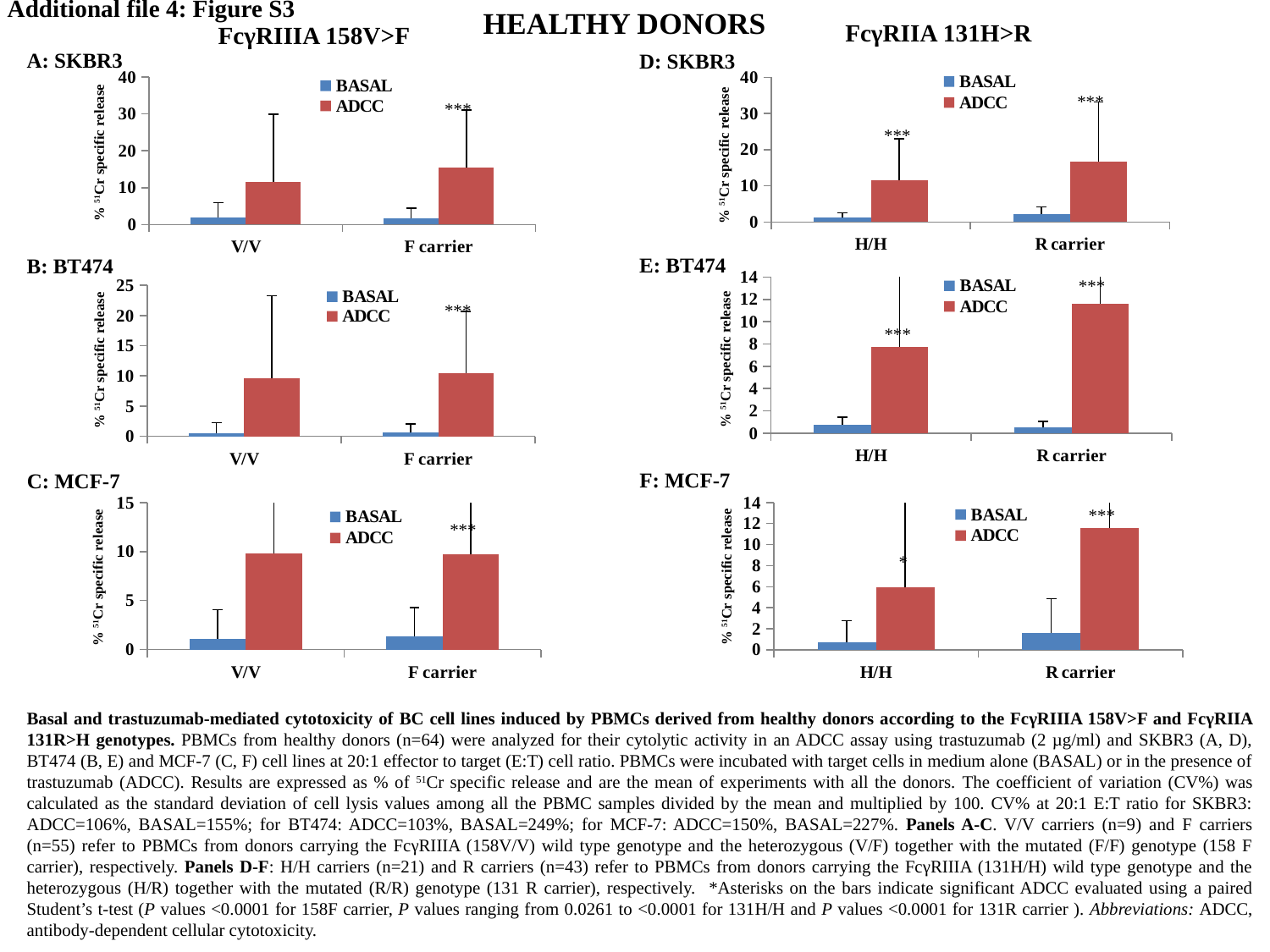

Additional file 4: Figure S3
HEALTHY DONORS
FcγRIIA 131H>R
FcγRIIIA 158V>F
A: SKBR3
D: SKBR3
### Chart
| Category | BASAL | ADCC |
|---|---|---|
| V/V | 2.0 | 11.555555555555555 |
| F carrier | 1.8 | 15.472727272727273 |
### Chart
| Category | BASAL | ADCC |
|---|---|---|
| H/H | 1.2857142857142858 | 11.523809523809524 |
| R carrier | 2.0930232558139537 | 16.58139534883721 |***
***
***
% 51Cr specific release
% 51Cr specific release
E: BT474
B: BT474
### Chart
| Category | BASAL | ADCC |
|---|---|---|
| H/H | 0.7142857142857143 | 7.761904761904762 |
| R carrier | 0.5348837209302325 | 11.627906976744185 |***
### Chart
| Category | BASAL | ADCC |
|---|---|---|
| V/V | 0.5555555555555556 | 9.666666666666666 |
| F carrier | 0.6 | 10.472727272727273 |***
***
% 51Cr specific release
% 51Cr specific release
F: MCF-7
C: MCF-7
### Chart
| Category | BASAL | ADCC |
|---|---|---|
| V/V | 1.1111111111111112 | 9.777777777777779 |
| F carrier | 1.3272727272727274 | 9.709090909090909 |
### Chart
| Category | BASAL | ADCC |
|---|---|---|
| H/H | 0.7142857142857143 | 5.904761904761905 |
| R carrier | 1.5813953488372092 | 11.581395348837209 |***
***
*
% 51Cr specific release
% 51Cr specific release
Basal and trastuzumab-mediated cytotoxicity of BC cell lines induced by PBMCs derived from healthy donors according to the FcγRIIIA 158V>F and FcγRIIA 131R>H genotypes. PBMCs from healthy donors (n=64) were analyzed for their cytolytic activity in an ADCC assay using trastuzumab (2 µg/ml) and SKBR3 (A, D), BT474 (B, E) and MCF-7 (C, F) cell lines at 20:1 effector to target (E:T) cell ratio. PBMCs were incubated with target cells in medium alone (BASAL) or in the presence of trastuzumab (ADCC). Results are expressed as % of 51Cr specific release and are the mean of experiments with all the donors. The coefficient of variation (CV%) was calculated as the standard deviation of cell lysis values among all the PBMC samples divided by the mean and multiplied by 100. CV% at 20:1 E:T ratio for SKBR3: ADCC=106%, BASAL=155%; for BT474: ADCC=103%, BASAL=249%; for MCF-7: ADCC=150%, BASAL=227%. Panels A-C. V/V carriers (n=9) and F carriers (n=55) refer to PBMCs from donors carrying the FcγRIIIA (158V/V) wild type genotype and the heterozygous (V/F) together with the mutated (F/F) genotype (158 F carrier), respectively. Panels D-F: H/H carriers (n=21) and R carriers (n=43) refer to PBMCs from donors carrying the FcγRIIIA (131H/H) wild type genotype and the heterozygous (H/R) together with the mutated (R/R) genotype (131 R carrier), respectively. *Asterisks on the bars indicate significant ADCC evaluated using a paired Student’s t-test (P values <0.0001 for 158F carrier, P values ranging from 0.0261 to <0.0001 for 131H/H and P values <0.0001 for 131R carrier ). Abbreviations: ADCC, antibody-dependent cellular cytotoxicity.
